# Molecular Determinants of Macrophage Polarization in Glioblastoma and Implications for Tumor Progression

**DOI:** 10.3390/cells15060508

**Published:** 2026-03-13

**Authors:** Xiao-Xiao Luo, Min Fu, Ben Zhao, Feng Yang, Yi-Zhou Liu, Xiao-Hong Peng, Shi-Yong Li, Gao-Feng Zhan, Ying-Jia Hu, Guang-Yuan Hu, Heng-Hui Cheng, Qian-Xia Li

**Affiliations:** 1 Department of Oncology, Tongji Hospital, Tongji Medical College, Huazhong University of Science and Technology, Wuhan 430030, Chinahu100810@tjh.tjmu.edu.cn (G.-Y.H.); 2 Department of Anesthesiology, Tongji Hospital, Tongji Medical College, Huazhong University of Science and Technology, Wuhan 430030, China; 3 Department of Public Health, School of Public Health, University of Pittsburgh, Pittsburgh, PA 15261, USA; 4 Department of Pathology, Tongji Hospital, Tongji Medical College, Huazhong University of Science and Technology, Wuhan 430030, China

**Keywords:** glioblastoma, macrophage, polarization

## Abstract

Glioblastoma (GBM) is a highly aggressive brain tumor with a complex tumor microenvironment (TME) that includes immune cell infiltration, notably macrophages. The role of macrophages in GBM progression is influenced by their polarization state, which can be either pro-inflammatory (M1) or immunosuppressive (M2). This study investigates the macrophage polarization in GBM, identifying key macrophage-related genes and their impact on tumor progression. Analysis of TCGA-GBM data revealed that macrophage infiltration correlates with poor prognosis, with 41 risk-associated genes identified. DSP dataset analysis highlighted 378 differentially expressed genes between CD68^+^ macrophages and GFAP^+^ controls, including immune-related genes like SPP1, CD74, and C3. Cross-validation with single-cell RNA-seq confirmed the expression of 9 key genes, with 7 genes being macrophage-specific. In vitro experiments using conditioned media from GBM cell lines demonstrated that GBM cells promote macrophage polarization towards an M2-like phenotype. Overexpression of CD74, CLEC7A, and IFI30 in macrophages further enhanced M2 polarization, which was associated with increased tumor-promoting functions, including enhanced invasion and reduced apoptosis in GBM cells. Together, these findings highlight the role of M2 macrophage polarization in promoting GBM progression and suggest that targeting macrophage polarization pathways may offer therapeutic potential.

## 1. Introduction

Glioblastoma (GBM) is one of the most aggressive and lethal forms of brain tumor [1], characterized by rapid growth, extensive infiltration into surrounding tissues, and resistance to conventional therapies such as surgery, radiotherapy, and chemotherapy [2,3,4]. The complex tumor microenvironment (TME) plays a critical role in GBM progression, influencing tumor growth, invasion, and response to treatment [5,6]. Immune cells, particularly macrophages, are key components of the TME and have been shown to either suppress or promote tumorigenesis depending on their polarization state.

Macrophages within the TME are commonly polarized into three primary phenotypes: M1, M2, and M0 macrophages [7,8]: M1 macrophages, which are pro-inflammatory and tumor-suppressive [9,10,11], and M2 macrophages, which are immunosuppressive and promote tumor progression [12,13]. M1 macrophages typically produce high levels of pro-inflammatory cytokines such as IL-1β, IL-6, and TNF-α, which are associated with antitumor immunity. In contrast, M2 macrophages, which can be further influenced by tumor-derived signals, generally secrete anti-inflammatory cytokines like IL-10 and TGF-β, fostering a tumor-promoting, immunosuppressive microenvironment [14]. M0 macrophages, considered the “resting” state, are a more neutral, unpolarized phenotype. While M0 macrophages do not have the distinct tumor-promoting or -suppressing functions of M1 or M2 macrophages, they are involved in the initial immune response and may transition into either M1 or M2 phenotypes under the influence of environmental signals, including those secreted by GBM cells [15]. The polarization of macrophages within the GBM microenvironment is influenced by various factors, including cytokines, growth factors, and soluble factors secreted by tumor cells. Recent studies have demonstrated that GBM cells secrete a variety of soluble factors that drive macrophage polarization toward the M2 phenotype, thereby facilitating immune evasion, tumor growth, and metastasis [16,17,18,19].

In the GBM TME, macrophages can originate from two distinct sources: microglia, the resident macrophages of the central nervous system (CNS) [20], and bone marrow-derived macrophages (BMDMs), which infiltrate the brain from the peripheral circulation in response to tumor signaling [21]. Microglia and BMDMs can exhibit similar polarization states (M1 or M2), but they differ in their origins, functional roles, and responses to the tumor environment. Microglia are involved in maintaining the homeostasis of the CNS [22], whereas BMDMs are recruited to the tumor site as part of the immune response to GBM [23]. Despite their similar functional phenotypes, microglia may have a more prominent role in immune surveillance and tissue repair, whereas BMDMs are more likely to be involved in promoting tumor progression through the secretion of immunosuppressive cytokines. The interaction between microglia and BMDMs in the GBM TME may have significant implications for the progression and treatment of the disease [15].

One of the key mechanisms through which GBM cells influence macrophage polarization is through the secretion of conditioned media (CM) that contain various cytokines, chemokines, and other signaling molecules. In particular, cytokines such as IL-6, IL-1β, and tumor necrosis factor (TNF-α) have been implicated in promoting M1 polarization [24,25,26], while factors like IL-4, IL-13, and prostaglandin E2 are known to promote M2 polarization. Recent research also indicates that specific surface markers such as CD74, CLEC7A, and IFI30 are upregulated during M2 polarization, playing pivotal roles in immune regulation and tumor progression [27,28,29,30,31,32].

In this study, we sought to investigate the role of macrophages in GBM progression by analyzing large-scale transcriptomic datasets, including data from TCGA and DSP, to identify macrophage-associated genes. We focused on the macrophage-related gene signatures and their correlation with GBM patient survival. Furthermore, we performed a series of functional validation experiments using conditioned media from GBM cell lines (U251 and U87) to examine how GBM-secreted factors influence macrophage polarization. Our study also explored the effects of overexpressing key M2 markers, CD74, CLEC7A, and IFI30, in macrophages and their potential to promote GBM progression by enhancing tumor cell invasion and survival.

Through these analyses, we identify a set of macrophage-associated genes that may serve as potential prognostic biomarkers for GBM and reveal how macrophage polarization toward the M2 phenotype promotes a tumor-supportive microenvironment. We propose that targeting macrophage polarization and the soluble factors that mediate this process could offer novel therapeutic strategies to enhance the efficacy of current GBM treatments.

## 2. Data and Methods

### 2.1. Datasets

Gene expression matrices and corresponding clinical information of glioblastoma (GBM) samples were obtained from The Cancer Genome Atlas (TCGA). Digital Spatial Profiling (DSP) data were collected from 18 patients with high-grade gliomas. Single-cell data: Single-cell RNA sequencing data comprising five GBM patient samples were retrieved from the Gene Expression Omnibus (GEO, accession number GSE256490).

### 2.2. Bulk Data Preprocessing

Raw count data were normalized and transformed into counts per million (CPM) expression matrices using the edgeR package in R 4.4.2. Differentially expressed genes were subsequently identified.

### 2.3. Immune Infiltration Analysis

Immune infiltration scores for each sample were estimated with CIBERSORT based on gene expression profiles. Pairwise correlations among different immune cell types were calculated. Genes showing strong correlations with macrophage infiltration were identified for downstream analyses.

### 2.4. Survival Analysis

Survival analyses were performed using the survival package in R. For each gene, patients were stratified into high- and low-expression groups according to the median expression value. Kaplan–Meier analysis and log-rank tests were conducted to identify genes significantly associated with overall survival.

### 2.5. Quality Control of Single-Cell Data

Single-cell data were processed following a standardized quality control pipeline. The DropletUtils 1.16.0 package was first used to identify expressed barcodes, and non-expressing barcodes were removed. Cells were further filtered based on the number of unique molecular identifiers (UMIs). Using the scater package 1.24.0, per-cell gene expression statistics were computed, and cells with >10% mitochondrial gene expression or <10% ribosomal gene expression were excluded. Doublets were identified and removed using DoubletFinder 2.0.3.

### 2.6. Preprocessing and Principal Component Analysis

After quality control, filtered datasets from all samples were integrated and normalized using the sctransform workflow in Seurat 4.4.0. Dimensionality reduction was then performed with RunPCA.

### 2.7. Cell Clustering

Principal components (PCs) with the highest variance were selected for downstream analyses. Clustering was carried out with the FindNeighbors and FindClusters functions in Seurat. Dimensionality reduction and visualization were performed using RunUMAP and RunTSNE.

### 2.8. Identification of Marker Genes

Differentially expressed genes for each cluster compared with all other cells were identified using the FindMarkers function in Seurat. The following thresholds were applied: log2 fold change (log2FC) ≥ 0.1, minimum expression fraction = 0.1, and adjusted *p*-value ≤ 0.05. The top 500 genes ranked by log2FC were defined as marker genes.

### 2.9. Cell Type Annotation

Cell type annotation was performed using SingleR, and further refined based on the identified marker genes. Annotated clusters were subsequently visualized using UMAP or t-SNE plots.

### 2.10. Target Gene Selection

Candidate genes were identified by integrating results across datasets. Genes that were associated with macrophage infiltration and prognosis in the TCGA dataset were intersected with differentially expressed genes derived from DSP data to obtain a set of target genes. Their expression patterns were then validated in the single-cell dataset, particularly within macrophage populations.

### 2.11. Cell Culture and Conditioned Medium Preparation

Human GBM cell lines (U251, U87; Wuxi Xinrun, Jiangsu, China, Cat# CH1258 and CH1079), normal human astrocytes (SVG p12; Fuheng, Shanghai, China, Cat# FH0563), and monocytic cell lines (THP-1, U937; Wuxi Xinrun, Cat# CH1137 and CH1037) were used. Cells were cultured in DMEM (Bio-Channel, Jiangsu, China, Cat# BC-M-005), MEM (Bio-Channel, Cat# BC-M-042), or RPMI-1640 (Bio-Channel, Cat# BC-M-023) supplemented with 10% fetal bovine serum (FBS; Hyclone, Logan, UT, USA, Cat# RL09136) and 1% penicillin/streptomycin (Biosharp, Beijing, China, Cat# BL505A).

For conditioned medium (CM) preparation, U251, U87, and SVG p12 cells were seeded into 6-well plates at 5 × 10^5^ cells/mL and cultured for 48 h. Supernatants were collected, centrifuged, and stored at −80 °C.

THP-1 and U937 monocytes were induced into M0 macrophages by treatment with 25 ng/mL PMA (Solarbio, Beijing, China, Cat# 227A023) for 24 h. The M0 macrophages were then cocultured with U251-, U87-, or SVG p12-derived CM for 72 h.

Overexpression plasmids for CD74, CLEC7A, and IFI30 (custom-constructed, verified by sequencing) were transfected into U937-derived macrophages using Lipofectamine 3000 (ThermoFisher, Waltham, MA, USA, Cat# L3000015). Vector-only transfection served as the control.

### 2.12. RT-qPCR

Total RNA was extracted using a commercial RNA extraction kit (Puhe, Wuxi, China, Cat# M004). Reverse transcription was performed with TRUEscript H Minus M-MuLV Reverse Transcriptase (Aidlab, Beijing, China, Cat# PC1703) and RNasin inhibitor (Aidlab, Beijing, China, Cat# RN3501). Quantitative PCR was conducted using SYBR Green qPCR Mix (Aidlab, Cat# PC3302) on an ABI 7500 Real-Time PCR system (Applied Biosystems, Waltham, MA, USA). GAPDH served as an internal control. Primer sequences were detailed in Supplementary Table S1.

### 2.13. Western Blotting

Cells were lysed with RIPA buffer (Beyotime, Shanghai, China, Cat# P0013B) supplemented with PMSF (Ruibio, Anhui, China, Cat# BP2655) and phosphatase inhibitors (Beyotime, Cat# S1873). Proteins were quantified using a BCA Protein Assay Kit (Biosharp, Beijing, China, Cat# BL521A). Proteins were separated by SDS-PAGE and transferred to PVDF membranes (Millipore, Darmstadt, Germany, Cat# IPVH00010). After blocking with 5% BSA, membranes were incubated overnight at 4 °C with the following primary antibodies: CD74 (Rabbit, ThermoFisher, Waltham, MA, USA, Cat# PA5-22113, 1:1000); CLEC7A (Rabbit, ThermoFisher, Cat# PA5-34382, 1:1000); IFI30 (Rabbit, Huamei Bio, Hubei, China, Cat# P13284, 1:1000); GAPDH (Mouse monoclonal, Proteintech, Hubei, China, Cat# 60004-1-Ig, 1:20,000). After washing, membranes were incubated for 1 h at 37 °C with HRP-conjugated goat anti-rabbit IgG (ZSGB-Bio, Beijing, China, Cat# ZB2301, 1:5000) or goat anti-mouse IgG (ZSGB-Bio, Cat# ZB2305, 1:5000). Bands were visualized using an ECL chemiluminescence kit (Beijing Dingguo, Beijing, China, Cat# ECL-0011).

### 2.14. Immunofluorescence

Macrophages were fixed with 4% paraformaldehyde (Beyotime, Cat#P0099), permeabilized with 0.1% Triton X-100 (Beyotime, Cat# P0096), and blocked with 5% FBS. Cells were incubated overnight at 4 °C with the following primary antibodies:

CD74 (Rabbit, ThermoFisher, Cat#PA5-22113, 1:200); CLEC7A (Rabbit, ThermoFisher, Cat#PA5-34382, 1:200); IFI30 (Rabbit, Huamei Bio, Cat#P13284, 1:200); CD163 (Mouse, Abcam, Cambridge, UK, Cat#Ab316218, 1:200). Cells were then incubated with secondary antibodies: goat anti-rabbit Cy3 (Proteintech, Cat# SA00009-2, 1:500) or goat anti-mouse FITC (Proteintech, Cat#SA00003-1, 1:500). Nuclei were counterstained with Hoechst 33258 (Beyotime, Cat# C1017), and images were acquired with an Olympus IX73 fluorescence microscope (Olympus, Tokyo, Japan).

### 2.15. Flow Cytometry

Cells were harvested, washed with PBS, and stained with the following fluorophore-conjugated antibodies: CD11b-FITC (BioLegend, Cat# 982614, 1:100); CD163-PE (BioLegend, Cat# 326505, 1:100). After incubation, samples were analyzed on a BD FACSVerse cytometer (BD, Franklin Lakes, NJ, USA), and the percentage of CD11b^+^CD163^+^ double-positive macrophages was determined.

### 2.16. Transwell Invasion Assay

The invasive capacity of U251 and U87 cells was assessed using Matrigel-coated Transwell chambers. Cells were seeded in serum-free medium into the upper chamber, while medium containing 10% FBS was added to the lower chamber. After 48 h, invaded cells were fixed with 4% paraformaldehyde, stained with crystal violet (Solarbio, Cat# G1063), and quantified under a microscope.

### 2.17. TUNEL Assay

Apoptosis was evaluated using a Calcein/PI cell viability and cytotoxicity detection kit (Beyotime, Cat# C2015S) in combination with Hoechst 33342 (Beyotime, Cat# C1027). Cells were stained according to the manufacturer’s protocol and imaged with an Olympus IX73 fluorescence microscope. The proportion of TUNEL-positive apoptotic cells was quantified.

### 2.18. Statistical Analysis

All experiments were performed in at least three independent biological replicates. Data are expressed as mean ± standard deviation (SD). Statistical significance between two groups was determined using Student’s *t*-test, while comparisons among multiple groups were analyzed by one-way ANOVA with post hoc tests. A *p* value < 0.05 was considered statistically significant.

## 3. Results

### 3.1. TCGA-GBM Data Analysis

A total of 172 GBM samples with clinical information (166 matched samples) from TCGA were included in the analysis. After normalization of raw counts data using the edgeR package, CPM expression matrices were generated for downstream analyses.

**Figure 1:**
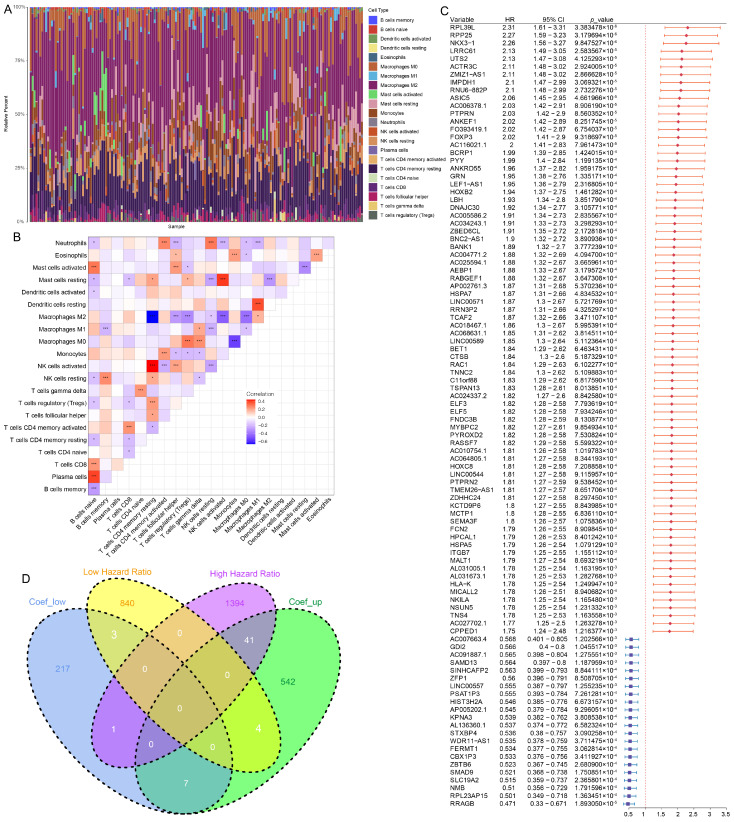
Identification of Prognostic Macrophage-Related Genes in GBM. (**A**) Immune cell composition across 166 TCGA-GBM samples estimated by CIBERSORT. Macrophage subsets (M0, M1, M2) show distinct infiltration patterns. (**B**) Correlation heatmap of immune cell subsets. Pearson correlation coefficients are color-coded; significant correlations are marked (*p* < 0.05). (**C**) Univariate Cox regression analysis of macrophage-related genes. Genes with HR ≥ 1.4 or ≤ 0.7 and *p* ≤ 0.05 were considered prognostically significant. (**D**) Venn diagram showing the overlap between macrophage-correlated genes and survival-associated genes. A total of 44 genes were identified, including 41 risk genes and 3 protective genes. * *p* < 0.05; *** *p* < 0.001.

Immune infiltration analysis was performed with CIBERSORT. The infiltration levels of macrophage subtypes (M0, M1, and M2) were extracted for each sample (Figure 1A). Correlation analyses between macrophage infiltration levels and gene expression profiles were conducted using Pearson correlation (Figure 1B). Based on the thresholds |correlation coefficient| ≥ 0.4 and FDR ≤ 0.05, a total of 827 macrophage-related genes were identified, including 599 positively correlated and 228 negatively correlated genes (Supplementary Table S2).

Survival analysis was subsequently performed. Genes were divided into high- and low-expression groups according to their median expression levels, and the associations with overall survival were assessed (Figure 1C). Using the criteria of *p* ≤ 0.05 and HR ≥ 1.4, a subset of prognostic genes was obtained.

Intersecting these prognostic genes with the macrophage-associated gene set yielded 44 overlapping candidates, including 41 risk-associated genes positively correlated with macrophage infiltration and 3 protective genes negatively correlated with macrophage infiltration (Figure 1D).

Intersecting these prognostic genes with the macrophage-associated gene set yielded 44 overlapping candidates. To clarify the prognostic orientation, we defined “High Hazard Ratio” as genes with a Hazard Ratio (HR) ≥1.4, indicating a significant association with increased mortality risk (shortened survival). Consequently, the intersection yielded 41 risk-associated genes (positively correlated with macrophage infiltration and HR ≥ 1.4) and 3 protective genes (negatively correlated with macrophage infiltration and HR < 1) (Figure 1D).

### 3.2. DSP Dataset Analysis

DSP data from 18 high-grade glioma patients were further analyzed, with a focus on CD68^+^ samples (*n* = 12) and their matched GFAP^+^ controls (*n* = 12). In addition, survival information was available for 12 samples, allowing comparisons between patients in the Dead (*n* = 6) and Alive (*n* = 6) groups.

**Figure 2:**
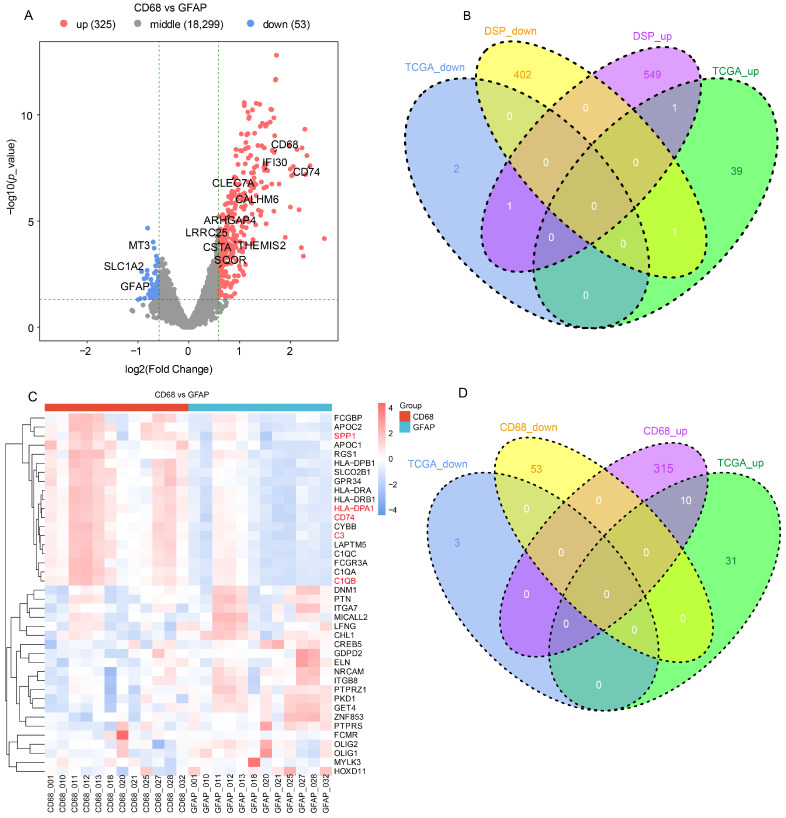
Identification of Macrophage-Enriched Prognostic Genes via DSP and TCGA Integration. (**A**) Volcano plot showing differentially expressed genes between CD68^+^ and GFAP^+^ samples in DSP data (FC ≥ 1.5, *p* < 0.05). A total of 378 genes were identified, with 325 upregulated (red) and 53 downregulated (blue). Key immune-related genes are labeled. (**B**) Venn diagram displaying overlap of differentially expressed genes between DSP (Alive vs. Dead) and TCGA survival groups. One gene was shared across all upregulated or downregulated gene sets. (**C**) Heatmap of the top differentially expressed genes between CD68^+^ and GFAP^+^ samples, highlighting enrichment of immune activation signatures in the CD68^+^ group. (**D**) Venn diagram showing the intersection of CD68^+^ vs. GFAP DEGs with TCGA-derived macrophage- and survival-associated genes, yielding 10 overlapping candidates for further analysis.

Differential expression analysis was conducted using edgeR. For the CD68 vs. GFAP comparison, 378 differentially expressed genes were identified (325 upregulated, 53 downregulated, FC ≥ 1.5, *p* < 0.05) (Figure 2A). In the updated volcano plot, we highlighted top-ranked genes from both compartments to ensure a balanced representation: immune-related signatures (e.g., SPP1, CD74, C3) were labeled in the CD68^+^-enriched (upregulated) side, while classical glial markers (e.g., GFAP, SLC1A2) were labeled in the GFAP^+^-enriched (downregulated) side (Figure 2A). For the Dead vs. Alive comparison, 954 genes were differentially expressed (549 upregulated, 402 downregulated) (Figure 2B). Volcano plots and heatmaps further underscored the distinct immune-related signatures in macrophage-enriched samples compared to the tumor-cell compartment (Figure 2C).

To further refine candidate genes, the overlap between DSP-derived DEGs and TCGA-identified macrophage- and prognosis-related genes was examined. The CD68 vs. GFAP intersection yielded 10 candidate genes for downstream analyses (Figure 2D).

### 3.3. Validation with Single-Cell RNA Sequencing Data

Single-cell RNA sequencing data from five GBM patient samples (GSE256490) were used to validate the candidate genes. Rigorous quality control was performed using DropletUtils, scater, and DoubletFinder (Supplementary Figure S1A–I). Cells with low UMI counts, high mitochondrial content (>10%), or identified as doublets were excluded. After preprocessing and normalization with the sctransform workflow in Seurat, dimensionality reduction was performed using PCA (Supplementary Figure S1J), and clustering was visualized via UMAP and t-SNE (Supplementary Figure S2A–C).

**Figure 3:**
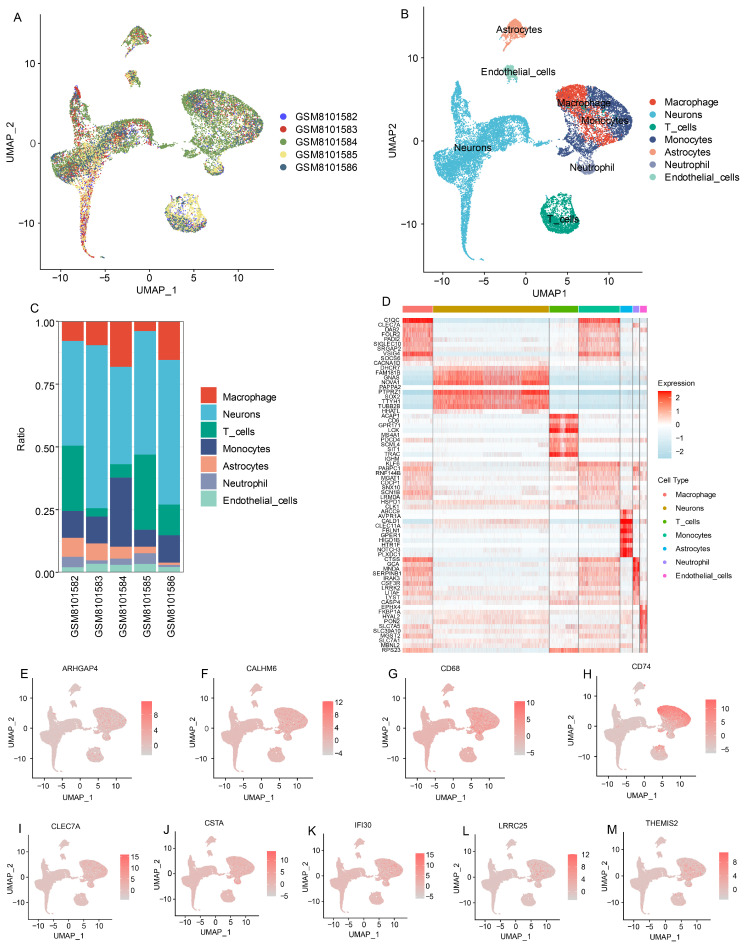
Single-Cell Validation of Candidate Macrophage-Associated Genes in GBM. (**A**) UMAP plot showing integration of single-cell RNA-seq data from five GBM patient samples (GSE256490). (**B**) Cell type annotation based on SingleR and marker gene expression, identifying macrophages, neurons, T cells, monocytes, astrocytes, neutrophils, and endothelial cells. (**C**) Proportional distribution of annotated cell types across individual samples. (**D**) Heatmap of selected marker genes across identified cell types, highlighting macrophage-specific gene expression patterns. (**E**–**M**) UMAP feature plots showing expression of 9 candidate genes (ARHGAP4, CALHM6, CD68, CD74, CLEC7A, CSTA, IFI30, LRRC25, THEMIS2), confirming their specific enrichment in macrophages.

Cell type annotation was carried out using SingleR combined with known marker genes. Macrophage populations were clearly identified among the clusters (Figure 3A–D). Expression analyses revealed that 9 out of the 10 candidate genes (all except SQOR) were specifically expressed in macrophages, confirming their biological relevance in the tumor immune microenvironment (Figure 3E–M).

### 3.4. Cross-Validation of Target Genes Across Datasets

**Figure 4:**
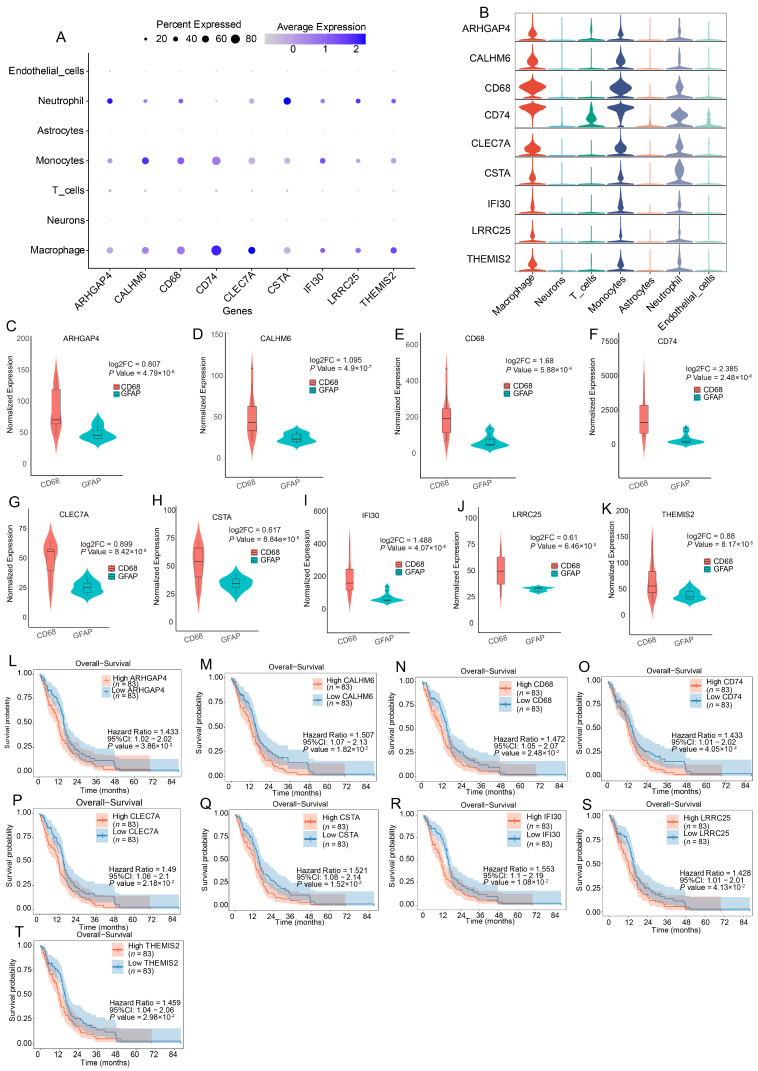
Validation and Prognostic Significance of Macrophage-Enriched Genes. (**A**) Dot plot showing average expression and percentage of cells expressing each gene across major cell types. Macrophage-specific enrichment is observed for all 9 candidate genes. (**B**) Violin plots displaying gene expression across different cell types, further confirming macrophage specificity. (**C**–**K**) Violin plots of gene expression in DSP data comparing CD68^+^ versus GFAP^+^ samples. All 9 genes are significantly upregulated in CD68^+^ macrophage-enriched regions. (**L**–**T**) Kaplan–Meier survival curves based on TCGA data showing that high expression of each gene is significantly associated with poorer overall survival, supporting their prognostic value.

The 9 macrophage-enriched genes were further validated in both DSP and TCGA datasets (Figure 4A,B). In the DSP data, all 9 genes were consistently upregulated in CD68^+^ samples compared to GFAP^+^ controls, as demonstrated by violin plots (Figure 4C–K). In TCGA, survival analysis confirmed that higher expression of these genes was significantly associated with poor overall survival, indicating that they function as risk-related prognostic biomarkers (Figure 4L–T).

### 3.5. Conditioned Medium–Induced Macrophage Polarization

**Figure 5:**
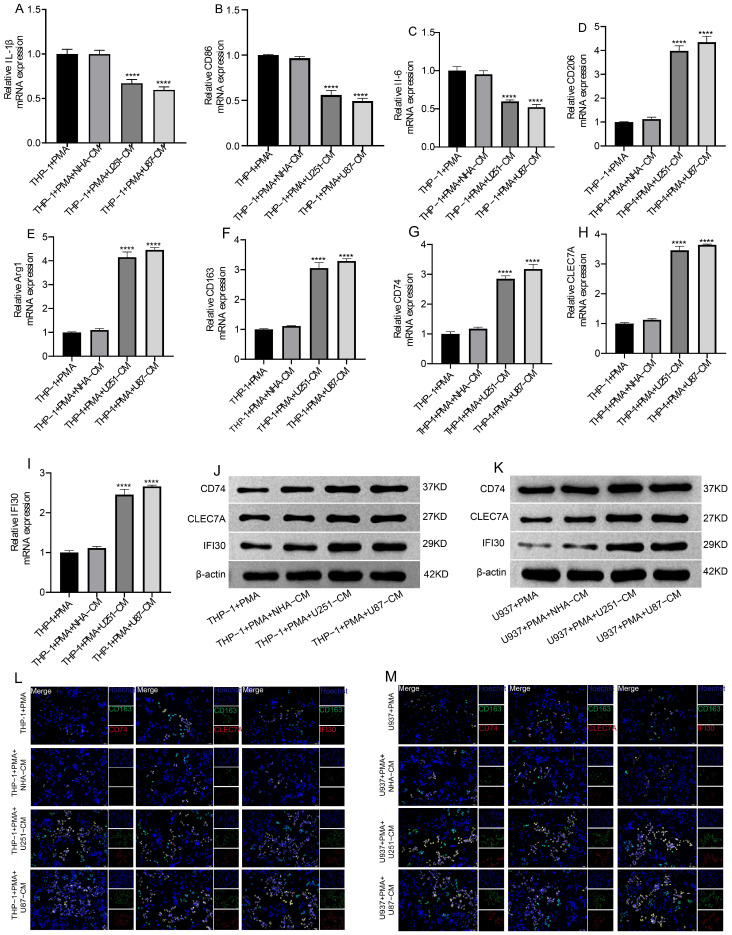
GBM-Derived Conditioned Media Induce M2-Like Polarization and Upregulation of Immunomodulatory Genes in Macrophages. (**A**–**I**) RT-qPCR analysis of macrophage markers after treatment with conditioned media (CM) from NHA, U251, and U87 cells. M1-associated genes (IL-1β, CD86, IL-6) were downregulated, while M2 markers (CD206, Arg1, CD163) and immunoregulatory genes (CD74, CLEC7A, IFI30) were significantly upregulated by U251-CM and U87-CM. (**J**,**K**) Western blot validation showing increased protein levels of CD74, CLEC7A, and IFI30 in THP-1–and U937–derived macrophages treated with U251-CM and U87-CM, but not NHA-CM. (**L**,**M**) Immunofluorescence staining confirming elevated expression of CD74, CLEC7A, and IFI30 in macrophages exposed to GBM-derived CM. Scale bars, 50 μm. Data are expressed as the mean ± standard deviation (SD) of three independent experiments, each conducted in triplicate. **** *p* < 0.0001.

To explore the effect of GBM cells on macrophage polarization, conditioned media (CM) derived from U251, U87, and normal human astrocytes (NHA) were applied to THP-1- and U937-derived macrophages. RT-qPCR results demonstrated that U251-CM and U87-CM treatment significantly decreased the expression of M1-associated markers (IL-1β, CD86, IL-6) (Figure 5A–C, Supplementary Figure S3A–C), while markedly increasing the expression of M2-related markers (CD206, Arg1, CD163) as well as immunomodulatory genes including CD74, CLEC7A, and IFI30 (Figure 5D–I, Supplementary Figure S3D–I). In contrast, NHA-CM treatment had no significant effect compared with the PMA-induced control.

At the protein level, Western blot analysis confirmed that CD74, CLEC7A, and IFI30 were strongly upregulated in macrophages exposed to U251-CM and U87-CM, whereas no difference was observed in the NHA-CM group (Figure 5J,K, Supplementary Figure S3J–O). Consistent with these findings, immunofluorescence staining revealed increased positive signals for CD74, CLEC7A, and IFI30 in macrophages treated with U251-CM and U87-CM, but not in the NHA-CM condition (Figure 5L,M, Supplementary Figure S3P,Q). Together, these results indicate that GBM cell–derived soluble factors promote macrophage polarization toward an M2-like, immunosuppressive phenotype.

### 3.6. Overexpression of CD74, CLEC7A, and IFI30 Promotes M2 Polarization

To further investigate the functional roles of CD74, CLEC7A, and IFI30, macrophages were transfected with overexpression plasmids for each gene. Western blot analysis verified successful transfection and protein overexpression (Supplementary Figure S4A–L).

**Figure 6:**
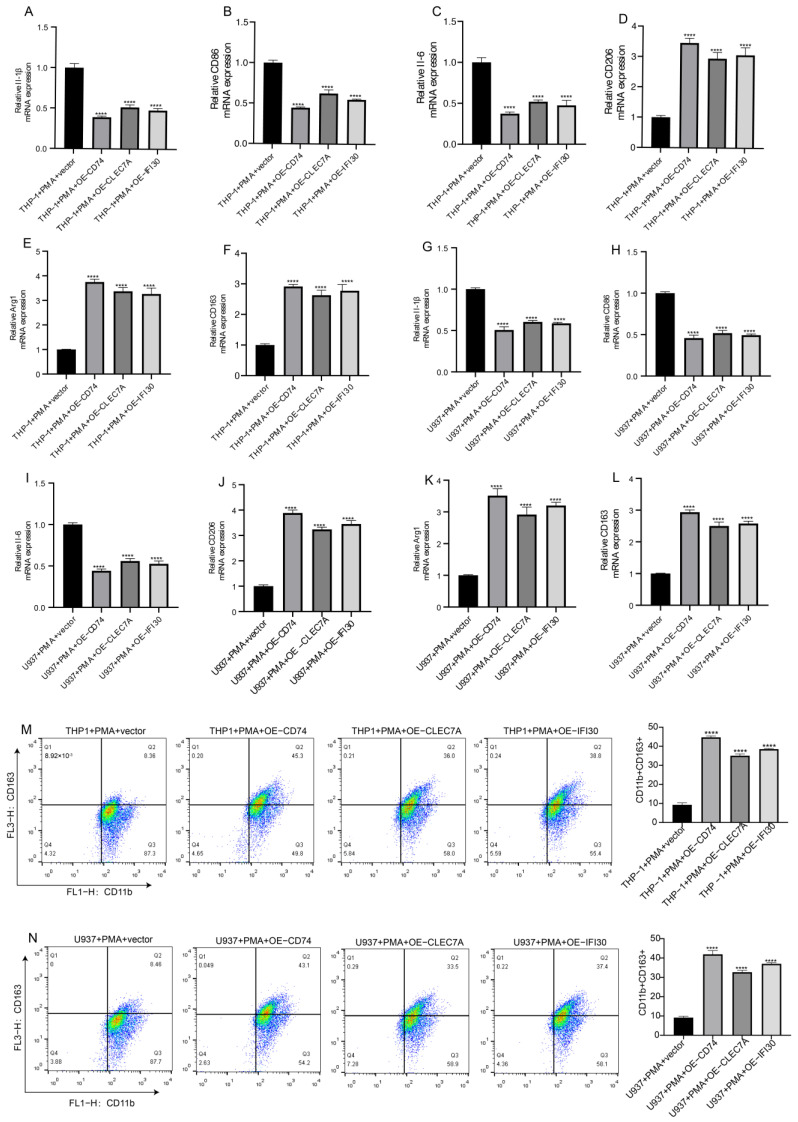
CD74, CLEC7A, and IFI30 Overexpression Promotes M2 Polarization in Macrophages. (**A**–**C**,**G**–**I**) RT-qPCR showing decreased expression of M1-associated genes (IL-1β, CD86, IL-6) in THP-1 and U937 macrophages following overexpression of CD74, CLEC7A, or IFI30. (**D**–**F**,**J**–**L**) Expression of M2-related markers (CD206, Arg1, CD163) is significantly increased in the overexpression groups compared to vector controls. (**M**,**N**) Flow cytometry analysis showing enhanced proportion of CD11b^+^CD163^+^ macrophages in THP-1 and U937 cells after CD74, CLEC7A, or IFI30 overexpression. Quantification is shown at right. Data are expressed as the mean ± SD of three independent experiments, each conducted in triplicate. **** *p* < 0.0001.

Subsequent RT-qPCR demonstrated that overexpression of CD74, CLEC7A, or IFI30 led to a pronounced downregulation of M1-related markers (IL-1β, CD86, IL-6), accompanied by significant upregulation of M2-associated markers (CD206, Arg1, CD163) (Figure 6A–L). Flow cytometric analysis further revealed that the proportion of CD11b^+^CD163^+^ macrophages was significantly increased in the overexpression groups compared with vector controls. These results provide strong evidence that CD74, CLEC7A, and IFI30 directly promote macrophage polarization toward the M2 phenotype (Figure 6M,N).

### 3.7. Overexpressed Macrophages Enhance GBM Malignant Progression

**Figure 7:**
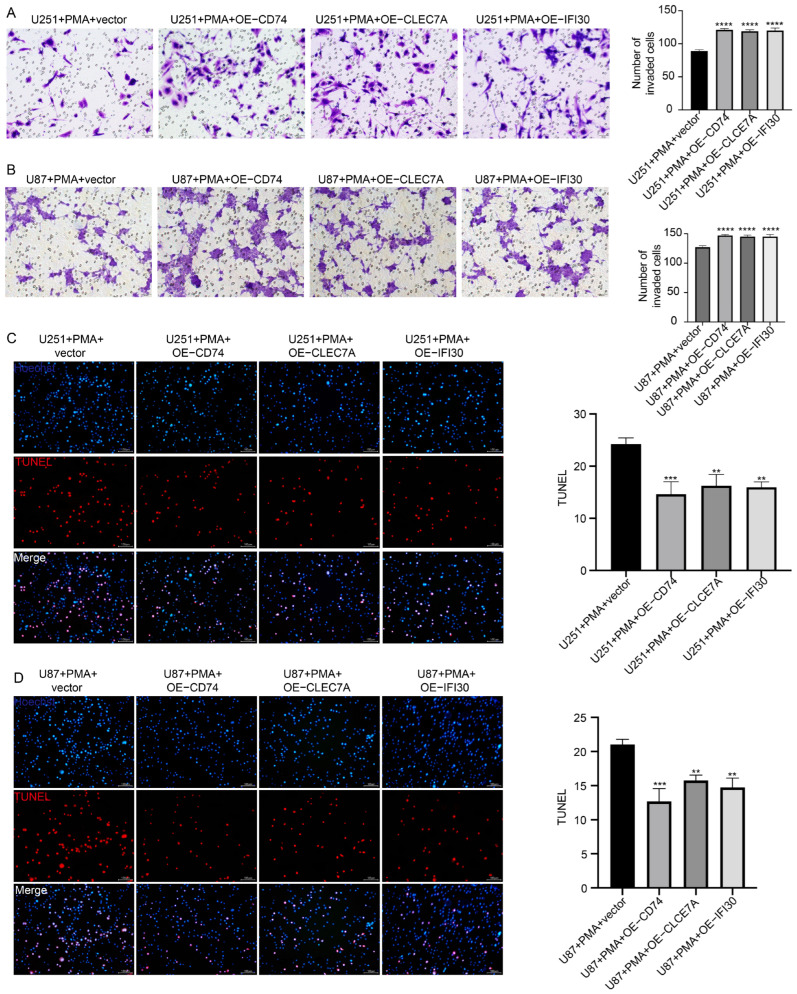
CD74, CLEC7A, and IFI30-Overexpressing Macrophages Enhance GBM Invasion and Inhibit Tumor Cell Apoptosis. (**A**,**B**) Transwell invasion assays showing that coculture with CD74-, CLEC7A-, or IFI30-overexpressing macrophages significantly promotes the invasive capacity of U251 and U87 glioma cells. Scale bars, 50 μm. Quantification is shown on the right. (**C**,**D**) TUNEL assays demonstrating reduced apoptosis in U251 and U87 glioma cells when cocultured with gene-overexpressing macrophages compared to vector controls. Scale bars, 100 μm. Bar graphs represent quantification of TUNEL-positive cells. Data are expressed as the mean ±SD of three independent experiments, each conducted in triplicate. ** *p* < 0.01; *** *p* < 0.001; **** *p* < 0.0001.

To assess the functional impact of macrophages overexpressing CD74, CLEC7A, or IFI30 on GBM progression, coculture experiments were performed. Transwell invasion assays revealed that both U251 and U87 glioma cells exhibited markedly enhanced invasive capacity when cocultured with gene-overexpressing macrophages compared with controls (Figure 7A,B).

In parallel, TUNEL assays showed a significant reduction in apoptosis rates of U251 and U87 cells upon coculture with CD74-, CLEC7A-, or IFI30-overexpressing macrophages (Figure 7C,D). Collectively, these findings demonstrate that macrophages enriched in CD74, CLEC7A, or IFI30 not only undergo M2 polarization but also foster a tumor-promoting microenvironment, thereby facilitating GBM invasion and survival.

## 4. Discussion

In this study, we explored the relationship between macrophage polarization and GBM progression, focusing on the roles of key macrophage-enriched genes, including CD74, CLEC7A, and IFI30. Our analyses using multiple datasets and experimental models suggest that GBM cells, through soluble factors, induce macrophage polarization toward an M2-like, immunosuppressive phenotype, which contributes to tumor progression [33,34]. Furthermore, overexpression of these macrophage-associated genes not only enhances M2 polarization but also facilitates GBM malignancy, promoting tumor invasion and survival [35,36].

In fact, these genes are known to play similar roles in various cancers by mediating immune modulation, promoting tumor progression, and influencing the metastatic potential of tumor cells. For example, in cervical cancer, CD74 has been shown to be involved in the regulation of immune responses, particularly by enhancing macrophage polarization towards the M2 phenotype [27,37]. Similarly, in breast cancer, CLEC7A (also known as Dectin-1) has been implicated in the immune response, where its activation supports the creation of an immunosuppressive TME, thus promoting tumor progression and metastasis [38,39]. IFI30, or gamma-interferon-inducible protein 30, has been found to regulate inflammation and immune responses across various cancer types, acting to suppress the host immune system and facilitating cancer cell survival [40,41].

The TCGA-GBM dataset analysis highlighted a significant association between macrophage infiltration and poor prognosis in GBM patients. Immune infiltration levels of macrophage subtypes (M0, M1, and M2) were examined, revealing that macrophages, particularly those of the M2 subtype, were positively correlated with several risk-associated genes. These findings underscore the role of macrophages in the tumor microenvironment and support previous studies indicating that M2 macrophages contribute to an immunosuppressive environment that supports GBM growth and metastasis [42,43]. The intersection of macrophage-related genes with prognostic markers further identified a set of 44 overlapping candidates, which were significantly correlated with survival outcomes, emphasizing the clinical relevance of macrophage infiltration in GBM prognosis.

Our study also examined how GBM cells influence macrophage polarization. Through conditioned media (CM) derived from U251 and U87 GBM cell lines, we demonstrated that GBM cells promote the polarization of macrophages toward the M2 phenotype, characterized by elevated expression of immunomodulatory genes such as CD74, CLEC7A, and IFI30. In contrast, conditioned media from normal human astrocytes (NHA) did not induce significant changes. This finding suggests that soluble factors secreted by GBM cells, rather than non-tumorigenic cells, drive the shift toward an immunosuppressive macrophage phenotype [44,45]. Crucially, the upregulation of these specific genes in response to GBM-CM suggests they may serve as the primary intracellular mediators through which the tumor microenvironment (TME) reprograms myeloid function.

To validate the functional significance of CD74, CLEC7A, and IFI30 as the downstream effectors of GBM-CM-induced polarization, we employed overexpression studies. While GBM-CM provides a complex stimulus involving multiple factors, our use of plasmid-mediated overexpression allowed for the mechanistic isolation of these three genes. Our results clearly showed that overexpression was sufficient to promote M2 polarization, mirroring the phenotypic shift observed with CM treatment. This confirms that the induction of CD74, CLEC7A, and IFI30 is not merely a correlative marker of CM stimulation but a causal driver of the M2-like state [46,47].

The functional impact of macrophage polarization on GBM progression was further assessed using coculture models. Transwell and TUNEL assays demonstrated that macrophages overexpressing CD74, CLEC7A, or IFI30 significantly enhanced tumor invasion and inhibited apoptosis. Given that GBM-CM significantly upregulates these three genes in macrophages, it can be logically inferred that the anti-apoptotic and pro-invasive effects of GBM-CM-treated macrophages are, at least in part, mediated through the activation of the CD74/CLEC7A/IFI30 axis. By isolating these genes, we provide a clearer molecular explanation for how GBM-secreted factors eventually foster an anti-apoptotic microenvironment. Although the total effect of CM may involve other redundant pathways, our data identify these three genes as critical functional nodes in this tumor-macrophage crosstalk, highlighting the role of M2-polarized macrophages in promoting tumor cell invasiveness [48,49].

In this study, the term ‘macrophages’ encompasses both resident microglia and bone marrow-derived macrophages (BMDMs). Although these two populations have distinct ontogenetic origins, they are known to exhibit functional convergence toward an M2-like phenotype within the glioblastoma microenvironment. Due to the inherent limitations of bulk RNA-seq data and the focus of our single-cell analysis on the collective tumor-associated macrophage (TAM) population, we focused on the collective macrophage population rather than performing separate sub-clustering of these lineages. Nevertheless, future investigations employing high-resolution lineage-tracing markers or advanced spatial deconvolution will be essential to further dissect the unique or redundant contributions of microglia and BMDMs to the CD74/CLEC7A/IFI30 axis [50,51,52,53]. Ultimately, targeting the macrophage-tumor interaction by modulating polarization remains a promising therapeutic strategy to attenuate the immunosuppressive TME in GBM. Further elucidation of the specific mechanisms by which these genes drive polarization and facilitate tumor-macrophage crosstalk will be critical for developing precision therapies aimed at reprogramming TAMs toward a pro-inflammatory, anti-tumor phenotype.

In conclusion, our findings highlight the essential role of macrophages in GBM progression and the potential of macrophage-related genes as therapeutic targets. By modulating macrophage polarization, we may be able to develop strategies that not only enhance immune response but also inhibit tumor growth and invasion, improving clinical outcomes for GBM patients.

## Data Availability

Most of the data sets used and/or analyzed during the current study are publicly available data from TCGA and GEO databases (GSE256490).

## Supplementary Materials

The following supporting information can be downloaded at: https://www.mdpi.com/article/10.3390/cells15060508/s1, Supplementary Table S1: Primer sequences; Supplementary Table S2. Supplementary Figure S1: Quality Control and Preprocessing of scRNA-seq Data from GBM Samples; Supplementary Figure S2: t-SNE Clustering of Single-Cell RNA-seq Data from GBM Samples; Supplementary Figure S3: Effect of GBM-Derived Conditioned Media on Macrophage Polarization; Supplementary Figure S4: Verification of CD74, CLEC7A, and IFI30 Overexpression in Macrophages.
