# Supplementary material for: Molecular Determinants of Macrophage Polarization in Glioblastoma and Implications for Tumor Progression
Source: Cells. 2026 Mar 13;15(6):508. doi: 10.3390/cells15060508 (PMC13025859; doi:10.3390/cells15060508)
Supplement: Supplementary file 1 [file cells-15-00508-s001.zip › Supplementary cells-4126465/Supplementary.docx]

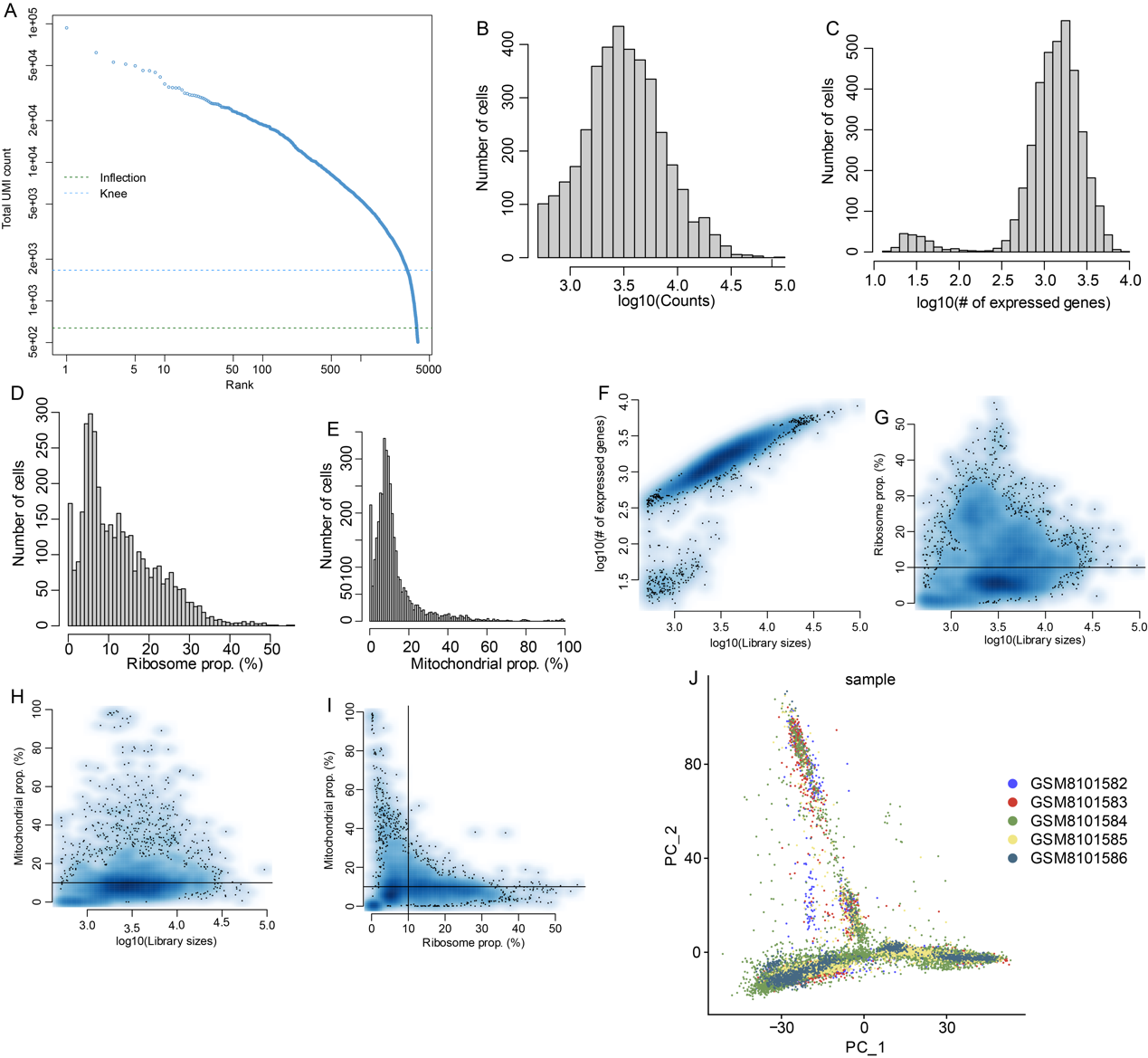


Supplementary Figure 1. Quality Control and Preprocessing of scRNA-seq Data from GBM Samples. (A) Barcode rank plot showing total UMI counts per cell. Inflection and knee points were used to define high-quality cells. (B–C) Distributions of log-transformed library sizes and number of detected genes per cell. (D–E) Histograms of ribosomal and mitochondrial transcript proportions. (F–G) Relationship between library size and gene count (F), and ribosomal percentage (G). (H–I) Scatter plots showing mitochondrial content relative to library size (H) and ribosomal content (I). Thresholds were applied to filter low-quality or stressed cells. (J) PCA plot illustrating batch-specific variation across five GBM samples (GSM8101582–GSM8101586) before batch correction.


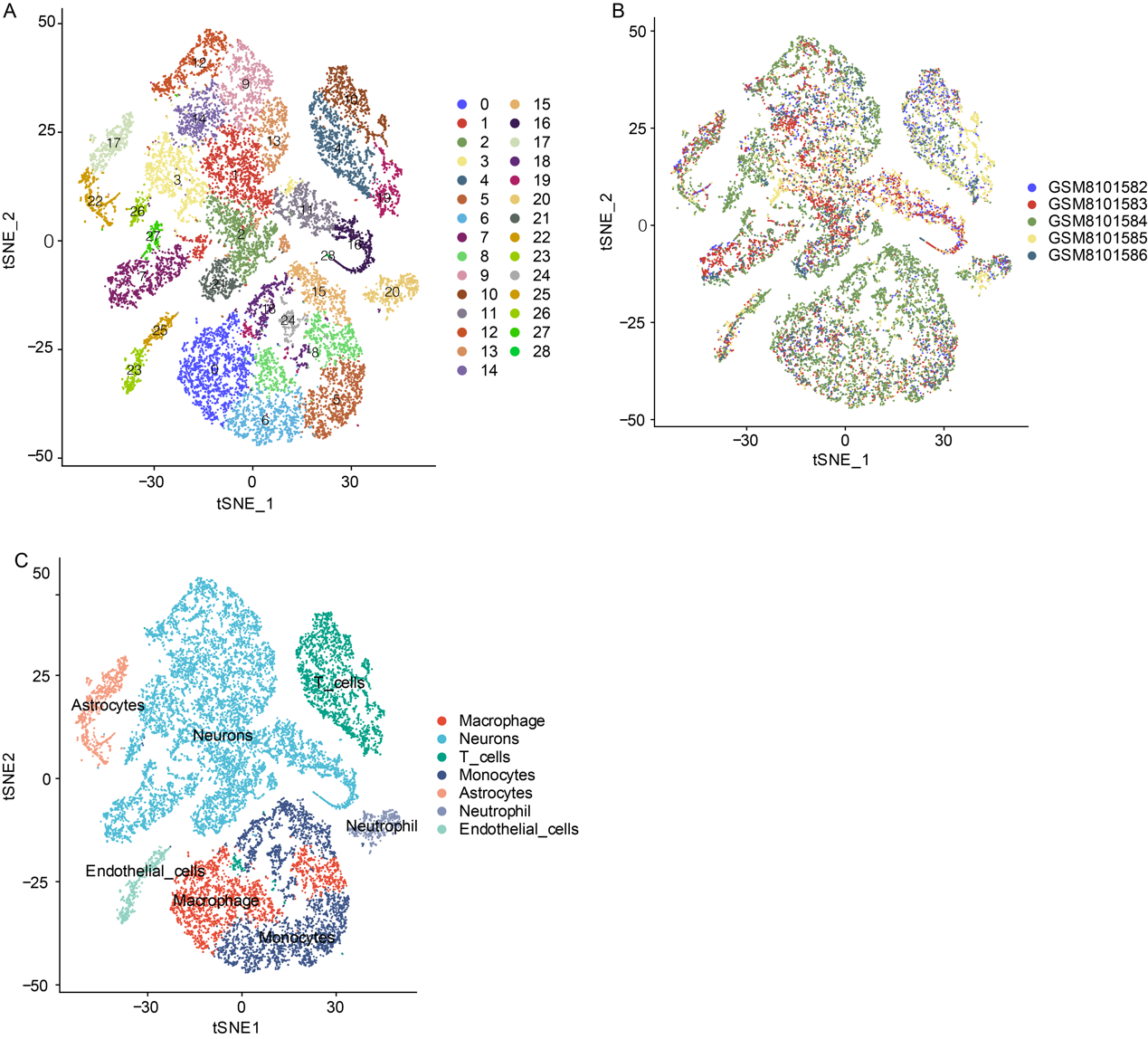


Supplementary Figure 2. t-SNE Clustering of Single-Cell RNA-seq Data from GBM Samples. (A) t-SNE plot showing clustering of cells based on transcriptomic profiles. Each cluster is color-coded, with cluster numbers annotated. (B) t-SNE plot showing batch-specific distribution of cells from five GBM patient samples (GSM8101582–GSM8101586). Each sample is represented by a unique color. (C) t-SNE plot with cell type annotation based on gene expression, identifying macrophages, neurons, T cells, monocytes, astrocytes, neutrophils, and endothelial cells.


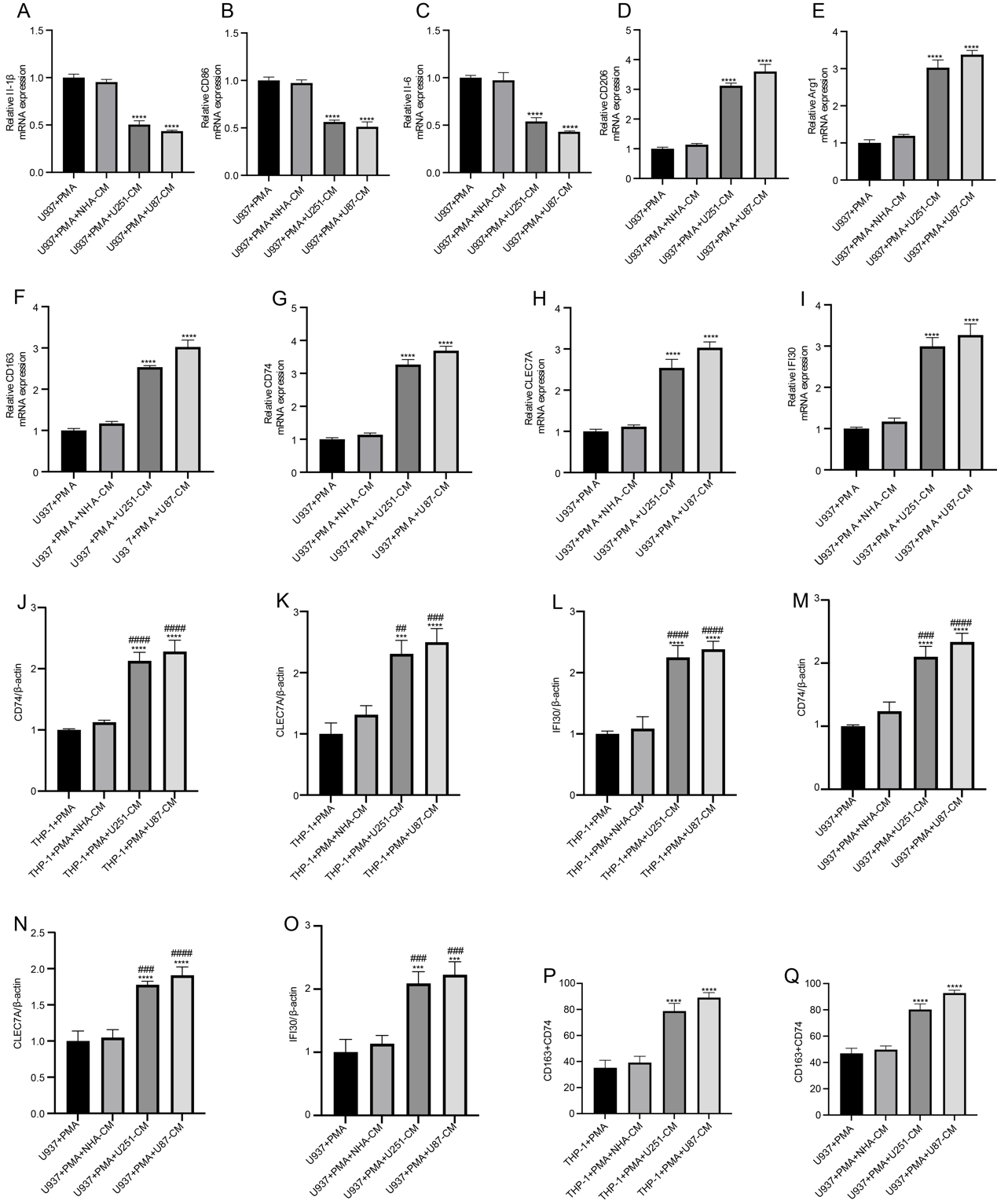


Supplementary Figure 3. Effect of GBM-Derived Conditioned Media on Macrophage Polarization. (A–C) RT-qPCR analysis showing decreased expression of M1-associated markers (IL-1β, CD86, IL-6) in macrophages treated with U251-CM and U87-CM, compared to the PMA-induced control. NHA-CM treatment had no significant effect. (D–I) RT-qPCR results showing increased expression of M2-related markers (CD206, Arg1, CD163) and immunomodulatory genes (CD74, CLEC7A, IFI30) in macrophages treated with U251-CM and U87-CM. No significant changes were observed in the NHA-CM group. (J–K) Western blot analysis confirming strong upregulation of CD74, CLEC7A, and IFI30 in macrophages exposed to U251-CM and U87-CM. No significant change was observed in NHA-CM-treated macrophages. (L–M) Immunofluorescence staining showing increased expression of CD74, CLEC7A, and IFI30 in macrophages treated with U251-CM and U87-CM, but not in NHA-CM-treated macrophages.


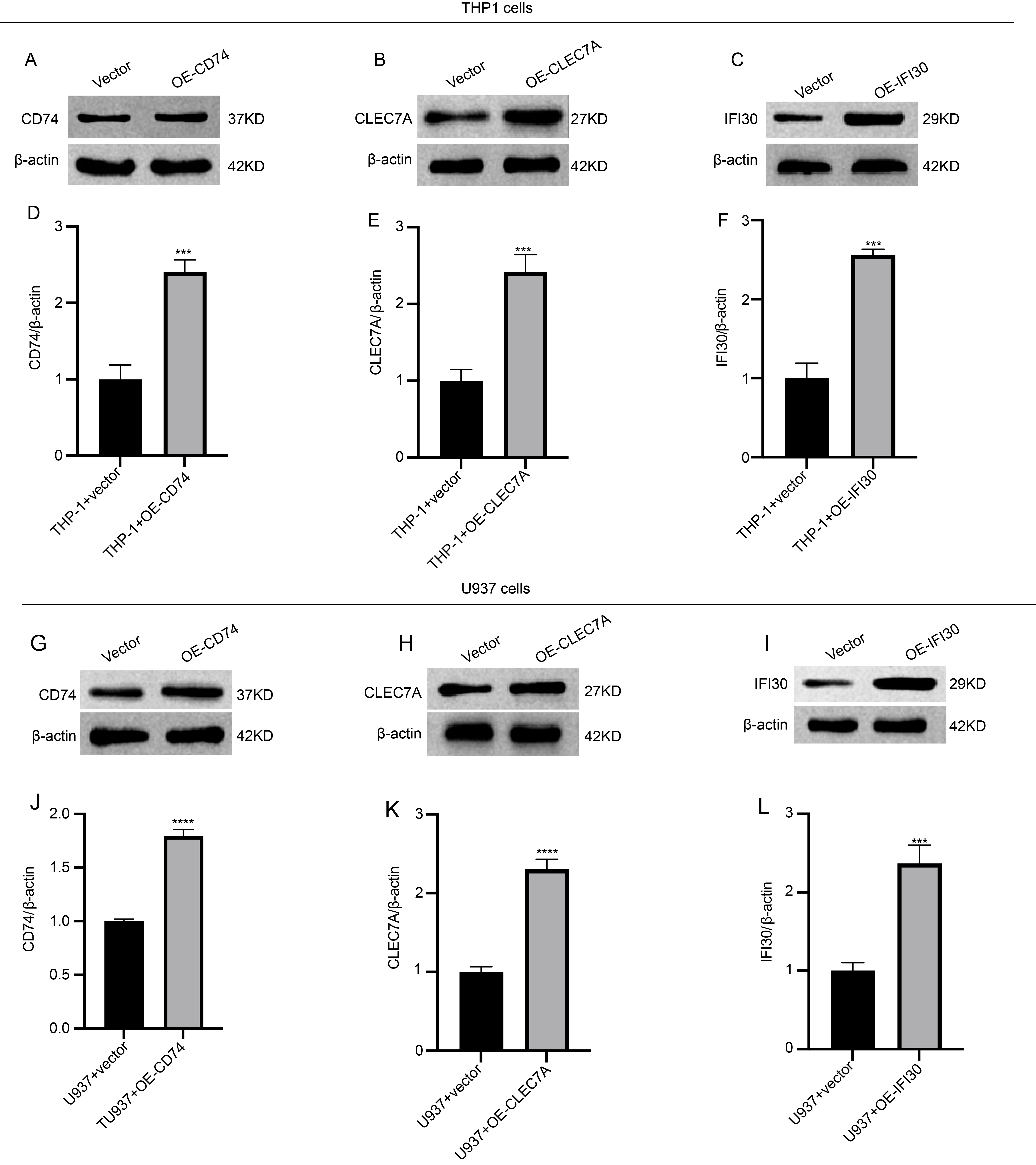


Supplementary Figure 4. Verification of CD74, CLEC7A, and IFI30 Overexpression in Macrophages. (A–C) Western blot analysis confirming successful overexpression of CD74 (A), CLEC7A (B), and IFI30 (C) in THP-1 macrophages transfected with overexpression plasmids. β-actin was used as a loading control. (D–F) Quantification of protein expression in THP-1 cells, showing significant upregulation of CD74 (D), CLEC7A (E), and IFI30 (F) following overexpression compared to vector controls. (G–I) Western blot analysis confirming overexpression of CD74 (G), CLEC7A (H), and IFI30 (I) in U937 macrophages. (J–L) Quantification of protein expression in U937 cells, demonstrating significant upregulation of CD74 (J), CLEC7A (K), and IFI30 (L) following overexpression compared to vector controls.

Supplementary Table 1. Primer sequences

| Primers | Sequence (5'-3') | PCR product size (bp) |
| --- | --- | --- |
| *Il-1β*（human）-RT-F | ATGGCTTATTACAGTGGCA | 138bp |
| *Il-1β*（human）-RT-R | GTAGTGGTGGTCGGAGATT |  |
| *Cd86*（human）-RT-F | ATACACGGTTACCCAGAA | 148bp |
| *Cd86*（human）-RT-R | CAGGGAATGAAACAGACA |  |
| *Il-6*（human）-RT-F | GGAGACTTGCCTGGTGAA | 195bp |
| *Il-6*（human）-RT-R | GCATTTGTGGTTGGGTCA |  |
| *Cd206*（human）-RT-F | AAGGTGACCCTACTATGT | 130bp |
| *Cd206*（human）-RT-R | GTAACTGGTGGATTGTCT |  |
| *Arg1*（human）-RT-F | CATAGGGATTATTGGAGC | 186bp |
| *Arg1*（human）-RT-R | TCATTAGGGATGTCAGCA |  |
| *Cd163*（human）-RT-F | TCCCGTCAGTCATCCTTTA | 158bp |
| *Cd163*（human）-RT-R | TCCCGGTATTGAATTTGGT |  |
| *Cd74*（human）-RT-F | TGTCGGGAAGATCAGAAGC | 190bp |
| *Cd74*（human）-RT-R | ACAGGAAGTAGGCGGTGGT |  |
| *Clec7a*（human）-RT-F | GTTCTTTCCAGCCCTTGT | 111bp |
| *Clec7a*（human）-RT-R | CAGTTGCCAGCATTGTCT |  |
| *Ifi30*（human）-RT-F | CACCGCTTGTCAATGTGA | 142bp |
| *Ifi30*（human）-RT-R | TCCTGTGCGTTTCCGTAG |  |
| *Arhgap4*（human）-RT-F | TTGGACCTCATGGACTGC | 172bp |
| *Arhgap4*（human）-RT-R | ACAAAGCCAAGGTTCTCG |  |
| *Calhm6*（human）-RT-F | TGGGCTGGATCTTGATAG | 154bp |
| *Calhm6*（human）-RT-R | TGCTCTGTGGCTTTACTT |  |
| *Csta*（human）-RT-F | TACCTGGAGGCTTATCTG | 182bp |
| *Csta*（human）-RT-R | ATTATCACCTGCTCGTAC |  |
| *Lrrc25*（human）-RT-F | CGGATGTGGACTGGAACG | 164bp |
| *Lrrc25*（human）-RT-R | CAGGTGGGCAAAGAAGGT |  |
| *Themis2*（human）-RT-F | CTTGAGGCTGTGGTGATG | 184bp |
| *Themis2*（human）-RT-R | TGAGGACGAGGTCTTTCA |  |
| *Gapdh*（human）-RT-F | GGAGCGAGATCCCTCCAAAAT | 197bp |
| *Gapdh*（human）-RT-R | GGCTGTTGTCATACTTCTCATGG |  |
